# Predicting Retention of American Indian and Alaska Native Research Participants in the NACC Dataset

**DOI:** 10.1002/alz70860_105639

**Published:** 2025-12-23

**Authors:** Kyle R Conniff, Josh D Grill, Daniel L Gillen

**Affiliations:** ^1^ University of Wisconsin‐Madison, Madison, WI, USA; ^2^ University of California, Irvine, Irvine, CA, USA; ^3^ The UC Irvine Institute for Memory Impairments and Neurological Disorders, Irvine, CA, USA; ^4^ The UC Irvine Institute for Alzheimer's Disease Research Center, Irvine, CA, USA

## Abstract

**Background:**

The number of American Indian and Alaska Native (AI/AN) older adults 65+ years old is growing. It is estimated that 1 in 3 AI/AN older adults will develop Alzheimer's disease or related dementias in their lifetime. With the number of AI/AN older adults expected to double by 2060, the number of older adults living with dementia is projected to increase four‐fold in the same time frame. Further, recent research has shown that AI/AN dementia‐research participants are retained at a lower rate than non‐Hispanic White participants. We sought to identify factors that predict retention of AI/AN participants as a vital step toward informing future research in potential areas of intervention.

**Method:**

We used logistic regression and 5‐fold cross‐validation to identify factors that maximize Area Under the Receiver Operating Characteristic Curve (AUC) for predicting retention to first follow‐up visit among AI/AN participants in the National Alzheimer's Coordinating Center Uniform Data Set. We defined retention to first follow‐up as a visit within 18 months of the initial visit. Over 500,000 first‐order models with 28 demographic and health‐related variables were considered.

**Result:**

For 220 AI/AN participants, the average AUC from most predictive model of retention was 0.76. This model consisted of baseline‐measured variables: 1) Evaluator's opinion of validity of participant's responses, 2) Educational attainment, 3) Living situation, 4) Is subject's vision normal?, 5) Is subject's hearing normal?, 6) Total number of medications reported, 7) any Motor function‐related comorbidities, 8) any other comorbidities (excluding CVD, motor, cognition, behavioral, sleep, mental health, TBI, diabetes, and cancer). The second‐place model with an average AUC differing by less than 0.0002 additionally identified 1) sex, 2) any CVD‐related comorbidities, 3) any reported difficulties with daily activities as predictive variables.

**Conclusion:**

The retention of AI/AN research participants likely requires removing barriers to participation, as is true for all participants. The variables identified suggest that future interventions should consider removing barriers related to living situation and comorbidities affecting daily functioning (such as vision, hearing, and motor function impairments). Future work will consider predictors for the overall population and how those factors differ from the factors for specifically AI/AN participants.

